# Supplementary material for: Geospatial Distributions of Lead Levels Found in Human Hair and Preterm Birth in San Francisco Neighborhoods
Source: Int J Environ Res Public Health. 2021 Dec 22;19(1):86. doi: 10.3390/ijerph19010086 (PMC8751210; doi:10.3390/ijerph19010086)
Supplement: Supplementary file 1 [file ijerph-19-00086-s001.zip › ijerph-1496282-supplementary.pdf]

Supplemental Materials:

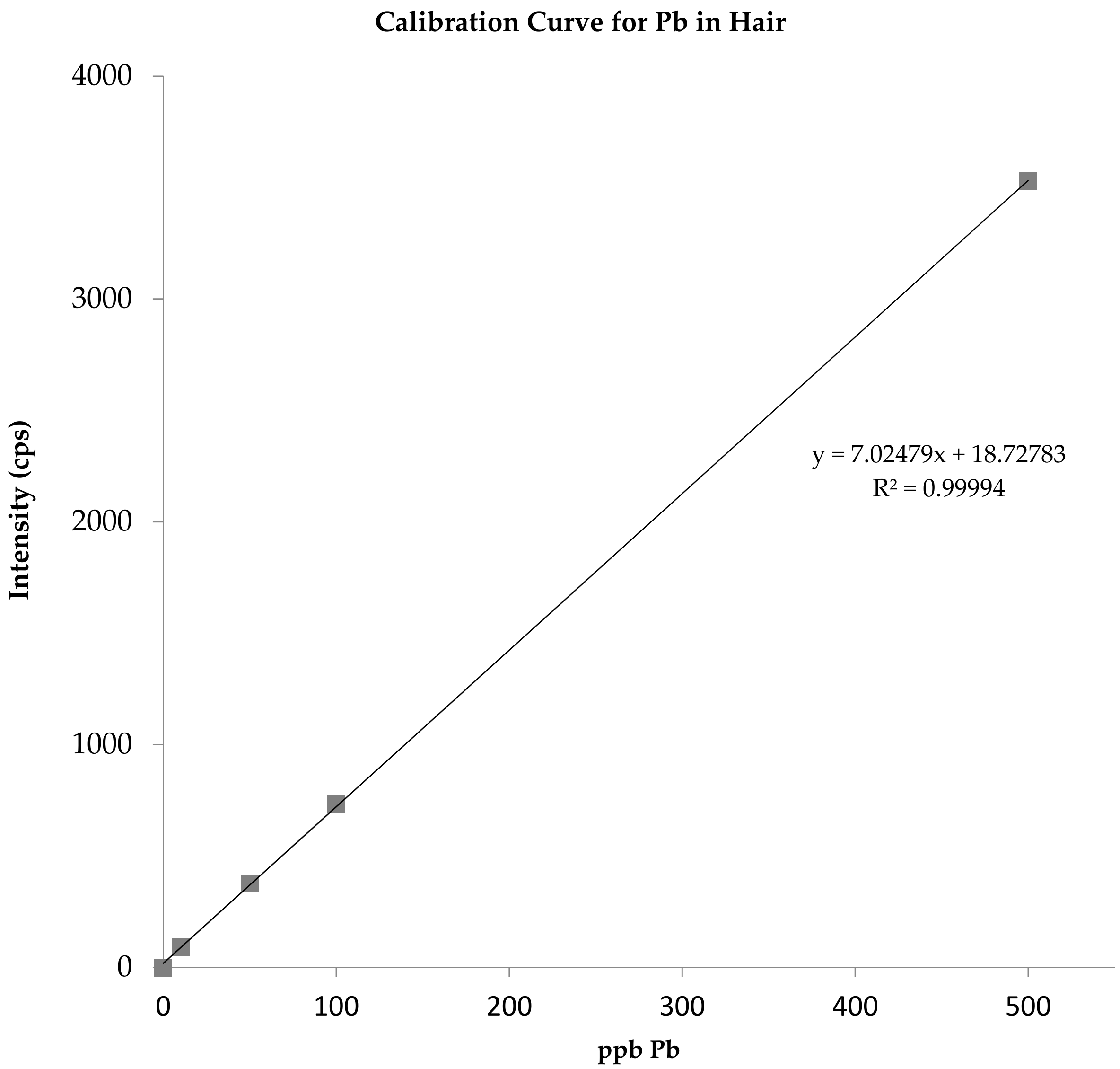

Supplemental Figure S1. Calibration Curve for Pb in Hair was set the following intensitiy - 1000, 200, 100, 50 ppb and Pb was highly detectable. R^2 value = 0.99994

Supplemental Table S1. Characteristics of Collected Hair samples -this represents the sample mean of the San Francisco population from participating hair salons that donated hair samples (n=108). ug/g = microgram of Pb per gram of hair and *Removed one extreme outlier during analysis.

	**Sample mean (±SD)**
Pb Concentration	4.7 ug/g (±2.5)
Preterm Birth	9.0% (±2.4)
Poverty	13.3% (±6.3)
Bachelor's Degree	34.3% (±7.7)
Gini Index	50.2% (±6.3)
African American Population	6.0% (±6.2)
Foreign-Born	32.3% (±11.3)

Supplemental Table S2. Unpaired t-test (two-sample t-test with unequal variances).

**Variable**	**Mean**	**Standard Error**	**Standard Deviation**	**[95% Confidence Interval]**		**p-value**
**Pb Concentration**	4.65463	0.2399968	2.49412	4.178864	5.130395	p<0.001
**Preterm Birth**	0.089537	0.0022725	0.0236167	0.085032	0.094042	p<0.001
**African American Population**	0 .059037	0.0060108	0.0624664	0.0471213	0.0709528	p<0.001
**Foreign-Born**	0 .3233611	0 .0109146	0 .1134278	0.3017242	0.344998	p<0.001
**Bachelor's Degree**	0 .3428982	0 .0074121	0. 0770293	0.3282044	0.3575919	p<0.001
**Poverty**	0 .1331111	0 .006027	0 .0626347	0.1211632	0.145059	p<0.001
**Gini Index**	0.5018519	0 .0060718	0 .0630998	0.4898153	0.5138885	p<0.001

Supplemental Table S3. Linear regression of continuous Pb concentration on preterm birth among hair samples from hair salons across 19 SF zip codes/neighborhoods (n=108).

Variable	**Model 1**		**Model 2**		**Model 3**		**Model 4**	
	β	SE	β	SE	β	SE	β	SE
**Pb**	0.003***	0.00	0.002***	0.00	0.001	0.00	0.001	0.00
**% Poverty**			0.123***	-0.03			0.058	-0.03
**% African/Black Population**					0.168***	0.03	0.135***	-0.03
Constant	0.077***	0.00	0.063***	0.00	0.075***	0.00	0.069***	0.00
R-square	0.076		0.181		0.241		0.258	

** p<0.05, ** p<0.01, ***p<0.001; Pb = lead; SE = standard error*

Supplemental Table S4. Linear regression of categorical Pb concentration on preterm birth among hair samples from hair salons across 19 SF zip codes/neighborhoods (n=108).

Variable	**Model 1**		**Model 2**		**Model 3**		**Model 4**	
	β	SE	β	SE	β	SE	β	SE
**Pb Level**								
0-2.9ug/g	*Ref*		*ref*		*ref*		*ref*	
3-5.7ug/g	0.011	0.01	0.007	0.00	0.007	0.01	0.007	0.00
5.8-17.2ug/g	0.014*	0.01	0.007	0.01	0.007	0.01	0.007	0.01
**% Poverty**			0.055	0.03			0.055	0.03
**% African/Black Population**			0.144***	0.03	0.175***	0.02	0.144***	0.03
Constant	0.081***		0.068***		0.074***		0.068***	
R-square	0.048		0.26		0.247		0.263	

** p<0.05, ** p<0.01, ***p<0.001; Pb = lead; SE = standard error*

Open source PTB Data:

The San Francisco Birth Data from 2012 Data contained within the article or supplementary material presented in this study are available at the City and County of San Francisco Department of Public Health- Maternal, Child & Adolescent Health. This brief report presents core tables and highlights of characteristics of these births, including health disparities, which reflect social inequalities, and opportunities for improvement of perinatal health in San Francisco. Reiter, R., & Chan, C. (2012). Birth Data San Francisco 2012; https://www.sfdph.org/dph/files/MCHdocs/Epi/Birth-Data-Summary-SF.pdf (accessed on 27, July 2019)**.** The San Francisco Birth Data from 2014 Data contained within the article or supplementary material presented in this study are available at the City and County of San Francisco Department of Public Health- Maternal, Child & Adolescent Health. This Data Brief summarizes characteristics of mothers in San Francisco who gave birth to a live infant in 2014. Stookey, J., & Chan, C. (2014). Data Brief: Live Births, 2014; https://www.sfdph.org/dph/files/MCHdocs/Epi/Updates12132016/SF-BirthsDataBrief2014-12-12-2016.pdf (accessed on 18, November 2019)

Open source Map Data:

The Census 2010 Tracts for San Francisco Data available in a publicly accessible repository that does not issue DOIs. Publicly available datasets were analyzed in this study. This data can be found here: [https://data.sfgov.org/d/rarb-5ahf?category=Geographic-Locations-and-Boundaries&view_name=Census-2010-Tracts-for-San-Francisco] (accessed on 12 October 2018). The Census 2010 blocks for San Francisco Data available in a publicly accessible repository that does not issue DOIs. Publicly available datasets were analyzed in this study. This data can be found here: [https://data.sfgov.org/d/2uzy-uv2r?category=Geographic-Locations-and-Boundaries&view_name=Census-2010-Blocks-for-San-Francisco] (accessed on 12 October 2018). The Bay area ZIP codes for San Francisco Data available in a publicly accessible repository that does not issue DOIs. Publicly available datasets were analyzed in this study. This data can be found here: [https://data.sfgov.org/Geographic-Locations-and-Boundaries/Bay-Area-ZIP-Codes/u5j3-svi6] (accessed on 12 October 2018). The Social Explorer San Francisco county Data available in a publicly accessible repository that does not issue DOIs. Publicly available datasets were analyzed in this study. This data can be found here: [https://www.socialexplorer.com/profiles/essential-report/san-francisco-county-california.html] (accessed on 30 November 2018). The EPA Data available in a publicly accessible repository that does not issue DOIs. Publicly available datasets were analyzed in this study. This data can be found here: [https://www.epa.gov/] (accessed on 2 September 2017).
